# Voyages in Vacation.—XVI

**Published:** 1889-01-26

**Authors:** 


### Voyages in Vacation.—XVI.

By One in Search of Restful Restoratives.

*(Continued from page 249.)*

FROM ST. PETERSBURG TO STOCKHOLM BY SEA.

The return journey by railway from Moscow to St. Petersburg proved uneventful, except that, owing to the presence of one of the Grand Dukes in the train, some of our party were unable to procure sleeping-berths. The time tables are made out between Moscow and St. Petersburg according to St. Petersburg time, and travellers would do well to bear this in mind, as in our case we were kept waiting at Moscow in a very close and overheated waiting-room for three-quarters of an hour owing to our ignorance of this fact. Arrived at St. Petersburg, we took the Hotel de France omnibus, and despite what Mr. Stead, of the *Pall Mall Gazette*, has written and said on Russian vehicles of this kind, we are bound to state in all fairness that it was one of the most comfortable omnibus rides we have ever had.

In the course of the day we had to call at the Guildhall or Mansion House of St. Petersburg, a conspicuous building situated in the Nevski Prospect, where the municipal authorities hold their sittings. The Lord Mayor, or Chief Magistrate, was not in St. Petersburg, but we obtained the address of his deputy, who proved a most courteous and obliging man. He accepted our invitation to see the *Victoria*, and duly arrived the following morning with three other friends or officials. They went over the whole of the vessel, manifesting much interest in the engines and indeed in all parts of the yacht, and afterwards (it being then 10 a.m.) determined that the only hospitality they could accept was a glass of sherry, of all drinks for such an hour.

We have to acknowledge our indebtedness to the municipal authorities of St. Petersburg for their co-operation and kindness in procuring most useful and complete information on various subjects in which we were interested. It was satisfactory to hear that the fame of the Lord Mayor of London had penetrated to St. Petersburg, and that the municipal authorities should have spontaneously declared that they always received such full information and ready compliance with requests forwarded to London that they were pleased to have an opportunity of reciprocating this courtesy. Indeed, we understood them to say their readiness to comply with our wishes was increased by the knowledge that this was the first occasion, or almost the first occasion, upon which any request for assistance of the kind had been made by an Englishman to the municipal authorities of St. Petersburg. In any case we gratefully recognise the completeness of the information we have received, and the thorough way in which the work has been carried out.

It was with feelings of satisfaction that we returned to the *Victoria* after our residence in Moscow, the comfort and airiness of the yacht, its companionship, and the freedom from the restrictions which we had had to submit to, being all very welcome. During our absence it appeared that the doctor with the captain had been analysing the water of the Neva, and that, after consideration, they had decided it was wholesome and good for drinking and cooking purposes. On this point there is room for at least two opinions, and Murray, for instance, declares "diarrhœa and dysentery are very prevalent, and strangers are very liable to suffer from those complaints, especially in the summer." It is difficult to say what causes the so-called "summer diarrhœa;" the water of the Neva has been blamed (more perhaps than it deserves to be); so has the position of St. Petersburg; so has the atmosphere; and so also have the vegetables, which last should be eaten sparingly at any rate. Murray then adds: "Travellers should drink as little as possible of the water of the Neva, for its disagreeble effects that sometimes felt even in tea." We pointed this out to the doctor, who said he had good scientific reason for believing Murray was wrong, and that in any case the water had been used and would be used without ill effects. We heard of no ill effects from the water if it was used, although before our departure for Moscow some of us had shown symptoms of mild dysentery. Whatever effects the internal application of the waters of the Neva may have upon those who drink them, there can be no doubt that Russians have good cause to be proud of their great river. "Magnificent!" is the exclamation of the stranger when he first sees this noble river, with its pure blue waters, which rush onward in one unceasing stream, unbroken by tide and unaffected by currents, until it reaches the sea. The true Russian will tell you that there is no ice in the world like Ladoga ice, that is ice procured from Lake Ladoga, the pure waters of which give birth to the River Neva.

It was a brilliant day when we weighed anchor and passed down the river and through the canal on our way to Cronstadt. We formed a merry party of friends, Mr. and Mrs. Dobson, the St. Petersburg correspondent of the *Times*, and others having come on board to accompany us there. The canal and the approach to St. Petersburg have been already described (see The Hospital, p. 103), but the sight is so unique in many respects that it in no way palled upon the beholders on the return journey. Mr. Dobson is one of the most diligent and notable scholars of Russia and the Russians. He has been through the Steppes, and many times to the Crimea, and his letters on the Trans-Continental Railway, which appeared in the *Times* during the latter part of last year, have added to his fame. We cannot, of course, say how far his recent imprisonment in an unsavoury dungeon, owing to the mistake made by a stupid official in the south of Russia, may have affected his views, but he gave very favourable accounts of his experiences, and produced, as some of the leading bankers and merchants also produced, much evidence in support of the opinion that Russia is, on the whole, a prosperous and wealthy country, and that its wealth and prosperity are rapidly on the increase.

Arrived at Cronstadt, we reluctantly parted from our friends, and, having passed the Custom House officials once more and taken the gunpowder and other things on board which had been left in their charge on our arrival, we proceeded into the Baltic, bound for Stockholm. It may be interesting to note that, although the inspection of the vessel and its discharge was this time comparatively perfunctory, it was noticeable that several more officials were in attendance than on the previous occasion—a fact which a wag on board put down to the credit of the *Victoria*, because it was proof positive that the liquor and viands provided commended themselves to the palates of the Russian officials. The power of assimilating alcohol in any and every form possessed by some Russians is astounding. Some, for example, seemed to have no difficulty in drinking gin, whisky, champagne, beer, and sherry, and even other things at the same sitting promiscuously, without any apparent regard as to the order of consumption. Possibly the climate may have something to do with the assimilating power here referred to, and although opinions may differ as to its admirable character, no one will deny that it probably excels that to be met with in any other nation. Up to this time we had had no rough weather, and the passage to Stockholm proved as calm as possible. The voyage is uninteresting enough until the archipelago of islands is entered, which extends from first to last for a distance of forty English miles, measuring from the commencement of Saltsjo, or the Bay of the Baltic, to Stockholm. Anything more picturesque or striking than the journey through these islands it is difficult to imagine. The scene is ever changing, and the variety of colour, of distance, and of outline, with the ever-varying characteristics of wood, valley, and homestead rivet the attention, and arouse sensations of pleasure and astonishment difficult to describe. Those who have seen the St. Lawrence with its thousand islands, and who have had the good fortune not merely to pass rapidly through them in a steamer, but to explore them at leisure, will find Stockholm and the bay of the Baltic with its innumerable islands a most charming residence in the summer, and one which is within easy distance of this country. The prices are reasonable, houses may be obtained without difficulty, the fishing is reported excellent, and the utmost facility of transit is afforded by the steam launches, which have been brought to a point of perfection hard to excel, and which may be had in any quantity for a small payment. About twelve miles from Stockholm, this, the only practicable approach for large vessels, is defended by two fortresses, one situated on a small island near Vaxholm, and the other on the Rindo. The artist would rejoice at the picturesque ruins of the round tower called the Fredriksborg, which used to constitute the only defence of the channel in more ancient times. So soon as the forts were passed we went down to breakfast, and on coming again on deck we found that the twelve miles had been traversed, and that Stockholm was in sight.

*(To be continued.)*

